# Prognostic value of the C-reactive protein–albumin–lymphocyte index for postoperative visual hallucinations following cardiac surgery

**DOI:** 10.3389/fpsyt.2026.1729831

**Published:** 2026-07-24

**Authors:** Haitham Abu Khadija, Abdalaziz Darwish, Mohammed Amer Kamel, Duha Najajra, Yahya Z. Fraitekh, Mohammad Masu’d, Wafiq Saleh Abdalkreem Othman, Omar Al-Haj, Mirna Mustafa, Ahmad Omar Darawsha, Hala Yaseen, Aya Abedallah Ibrahim, Yazan Hamdan, Yasmine Abu Khadija, Hasan Alkhatib, Helmi Mahmoud Tamimi, Moath Nairat, Idiberto José Zotarelli Filho, Nizar Abu Hamdeh, Mohammad Alnees

**Affiliations:** 1 Heart Center, Kaplan Medical Center, Rehovot, affiliated with the Hebrew University, Jerusalem, Israel; 2 Palestinian Clinical Research Center, Bethlehem, Palestine; 3 Department of Medicine, Faculty of Medicine and Allied Medical Sciences, An-Najah National University, Nablus, Palestine; 4 Department of Medicine, Faculty of Medicine, Al-Quds University, East Jerusalem, Palestine; 5 Clinical Research Program, Faculty of Graduate Studies, An-Najah National University, Nablus, Palestine; 6 Department of Anesthesia, An-Najah National University Hospital, Nablus, Palestine; 7 Department of Medical Science, Faculty of Medicine and Health Sciences, Hebron University, Hebron, Palestine; 8 Department of Cardiac Surgery, Al Makassed Hospital, Jerusalem, Palestine; 9 Department of Cardiothoracic Surgery, An-Najah National University Hospital, Nablus, Palestine; 10 UNESP - São Paulo State University, Institute of Biosciences, Humanities and Exact Sciences (Ibilce), Postgraduate Program in Food, Nutrition and Food Engineering, São Paulo, Brazil; 11 ABRAN - Brazilian Association of Nutrology, Catanduva, São Paulo, Brazil; 12 Harvard Medical School Postgraduate Medical Education, Global Clinical Scholars Research Training program, Boston, MA, United States

**Keywords:** CALLY index, cardiac surgery, inflammation, neuropsychiatric complications, nutrition, visual hallucinations

## Abstract

**Background:**

Visual hallucinations (VH) are distressing and clinically relevant after cardiac surgery. C-reactive protein–albumin–lymphocyte (CALLY) index reflects systemic inflammation, nutrition, and immune competence. This study evaluated whether preoperative CALLY predicts 7-day postoperative VH.

**Methods:**

In a prospective multicenter cohort (10 cardiac centers; September 2022 to May 2025), adults undergoing coronary or valve surgery were followed through day 7. Preoperative CALLY was calculated from routine laboratory measurements. VH discrimination was assessed using receiver operating characteristic (ROC) analysis, with the optimal cut-off by Youden’s index. Time-to-event analyses employed Cox regression with censoring at day 7, modeling CALLY continuously via restricted cubic splines (with 3 knots at the 10th/50th/90th percentiles) and categorically using a data-driven cut-off. Multivariable models adjusted for age, sex, body mass index (BMI), European System for Cardiac Operative Risk Evaluation II (EuroSCORE II), surgery type, cardiopulmonary bypass (CPB) time, left ventricular ejection fraction (LVEF), and postoperative ventilation hours.

**Results:**

Among 1,272 patients (150 VH events), the optimal CALLY cut-off was 1.12, separating Low (n=524) and High (n=748) groups. Kaplan–Meier curves diverged early (log-rank p<0.001). High vs. Low CALLY was associated with reduced VH risk (crude HR 0.39, 95% CI 0.28–0.54; adjusted HR 0.38, 95% CI 0.27–0.53; both p<0.001). When modeled continuously, CALLY demonstrated a non-linear association with VH risk (spline terms: HR 0.58 and 2.08; p=0.003 and p=0.023, respectively). Subgroup analyses showed broadly consistent associations, with no statistically significant effect modification by age, sex, diabetes, hyperlipidemia, or surgery type (all p for interaction >0.05).

**Conclusions:**

A lower preoperative CALLY index identifies cardiac-surgery patients at higher risk of VH and offers a practical tool for neuropsychiatric risk stratification.

## Introduction

Cardiac surgery, including coronary artery bypass grafting (CABG) and valve surgery, remains the mainstay of treatment for serious cardiovascular disease (1). In high-income countries, annual surgical volumes reach approximately 120–130 per 100,000 population (1–3). Despite improvements in surgical safety and outcomes, these procedures continue to carry risk of postoperative neuropsychiatric complications, including delirium, cognitive dysfunction, and hallucinations.

Hallucinations are sensory perceptions that occur in the absence of external stimuli and may include visual or auditory phenomena. They are increasingly recognized after major cardiac surgery (4). In a prospective study, 21.9% of patients developed postoperative hallucinations within the first four days, compared with only 4.1% in a matched cardiology outpatient control group, with visual phenomena being predominant (5). Importantly, while many showed concurrent delirium, most hallucinations occurred independently, indicating distinct pathophysiological pathways (5, 6). Clinically, visual hallucinations (VH) warrant focused attention because they are distressing postoperative perceptual disturbances that may complicate recovery, increase patient and family distress, and contribute to greater postoperative morbidity, prolonged hospitalization, higher healthcare costs, and poorer long-term outcomes (5, 7). Although VH may occur in the context of delirium, they are not synonymous with delirium and may also occur without the broader disturbances in attention, awareness, and cognition required for delirium diagnosis. Therefore, prediction models developed for postoperative delirium may not fully capture the risk of VH as a distinct clinical outcome. A practical preoperative biomarker-based tool for VH risk stratification may help identify vulnerable patients who could benefit from closer postoperative monitoring and early supportive measures.

The C-reactive protein–albumin–lymphocyte (CALLY) index is an emerging biomarker that integrates systemic inflammation, nutritional status, and immune competence, respectively (8). Accumulating literature has highlighted the prognostic utility of the CALLY index across diverse clinical contexts, particularly in oncology and critical illness (9, 10). Consistently, lower CALLY index values have been associated with adverse outcomes, including reduced survival and increased morbidity (11–13). Its prognostic value may reflect the combined effects of systemic inflammation, malnutrition, and impaired immunity during the postoperative period (8). In the setting of cardiac surgery, both inflammation and nutritional decline are common perioperative concerns. Thus, the CALLY index may serve as a promising predictor of postoperative hallucinations and related neuropsychiatric morbidity (14). To date, no study has examined the prognostic value of the CALLY index for hallucinations after cardiac surgery. We therefore aimed to evaluate its predictive role and establish an optimal cut-off for this population.

## Materials and methods

### Study design, setting, and population

This prospective cohort study was conducted across 10 cardiac surgery centers in the West Bank of Palestine, with patient recruitment and data collection performed between September 2022 and May 2025. It was implemented as part of the Visual and Auditory Hallucinations Following Cardiac Surgery (VAACS) study, an ongoing multicenter investigation of neuropsychiatric outcomes following cardiac procedures (https://www.isrctn.com/ISRCTN18368253). Participating centers included Ibn Sina Specialist Hospital, Specialized Arab Hospital, An-Najah National University Hospital, Al-Razi Hospital, Al-Mezan Hospital, Al-Ahli Hospital, Arab Society for Rehabilitation, Palestine Medical Complex, Nablus Specialty Hospital, and Al-Makassed Hospital.

Eligible participants were adult patients undergoing isolated coronary artery bypass grafting or isolated valve surgery at one of the participating centers during the study period, with available preoperative CRP, serum albumin, and lymphocyte measurements obtained within seven days before surgery and postoperative follow-up for VH assessment through day 7. Patients were excluded if they had conditions that could substantially alter the biological components of the CALLY index or compromise reliable outcome assessment, including hematologic disorders, active infection, active malignancy, severe malnutrition, systemic steroid therapy, early postoperative mortality before outcome assessment, documented psychotic disorders, missing or refused follow-up, combined CABG and valve procedures, other cardiac surgical procedures, age<18 years, cognitive or language barriers precluding reliable questionnaire administration, alcohol-use-related concerns, or relevant neurologic disorders.

**Figure 1:**
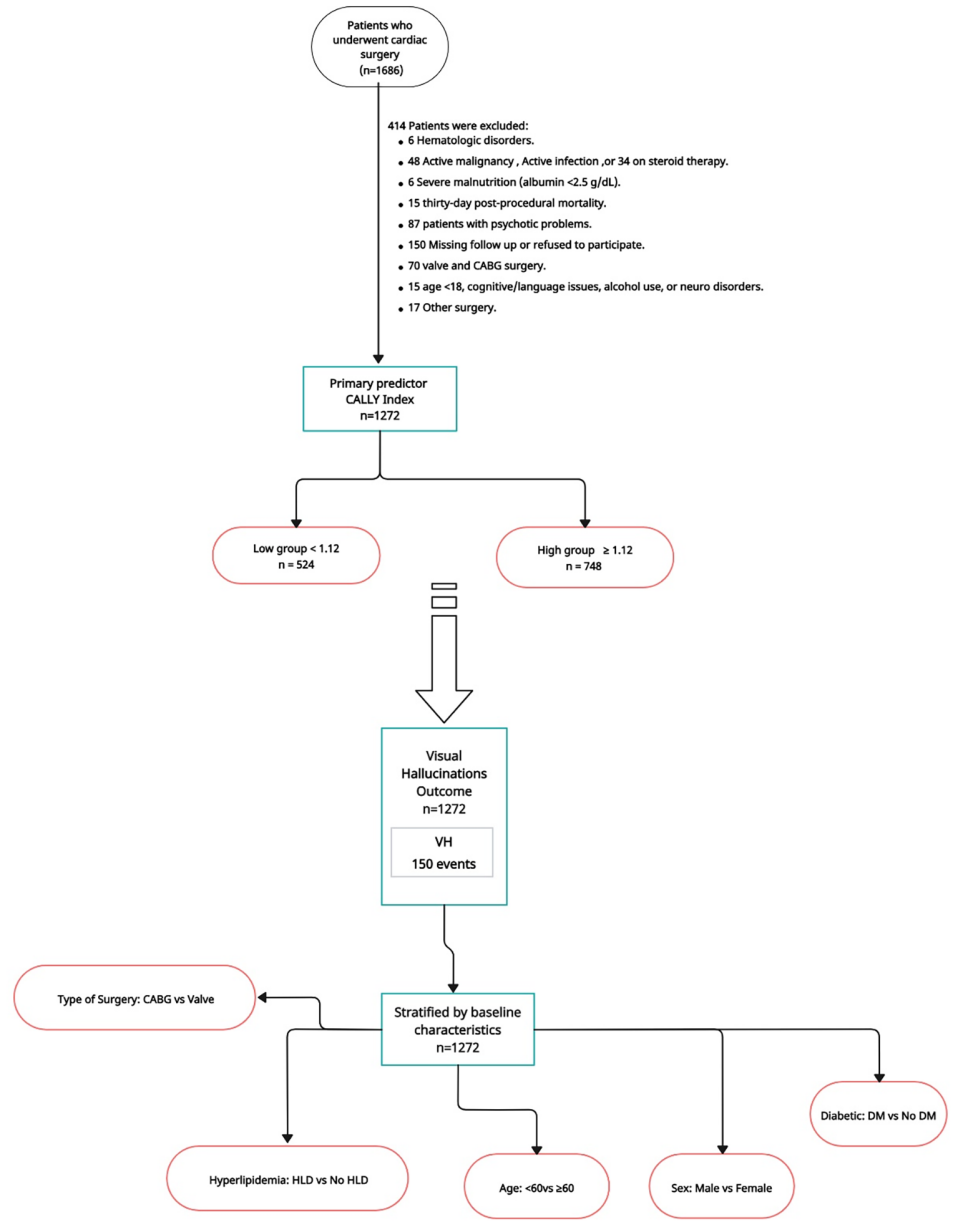
Flow chart.

Out of 1,686 patients who underwent cardiac surgery, 414 were excluded due to hematologic disorders, active infection, malignancy, severe malnutrition, early mortality, psychotic problems, missing follow-up, valve/CABG procedures, or other exclusion criteria. These criteria were predefined and applied to ensure the validity of the CALLY index by removing conditions known to significantly alter its biological components, including hematologic disorders, active infection, malignancy, and severe malnutrition. Importantly, exclusions were not based on outcome status. The final cohort included 1,272 patients, in whom the CALLY Index was the primary predictor. Patients were categorized into Low CALLY (<1.12, n=524) and High CALLY (≥1.12, n=748) groups. The primary outcome was visual hallucinations (VH), observed in 150 cases as shown in **Figure 1**.

### Data collection instruments

Hallucinations were assessed using the Questionnaire for Psychotic Experiences (QPE), a validated instrument that systematically evaluates hallucinations (15, 16). The QPE has demonstrated robust psychometric properties, including internal consistency across subscales, with Cronbach’s α values reported between 0.75 and 0.90, and has been validated in psychiatric and medical populations as a measure of psychotic symptoms (15–17). To ensure standardization, research staff underwent structured training sessions led by consultant psychiatrists, focusing on accurate symptom recognition, standardized interview conduct, and consistent administration of the QPE across participating centers before administering the questionnaire. Clinical and demographic covariates were extracted from electronic medical records (EMRs) using standardized case-report forms. Data collection was conducted prospectively during the study period (2022–2025).

### CALLY index

The CALLY index is a novel indicator derived from routine lab measures, calculated as the product of serum albumin and lymphocyte count divided by the C-reactive protein (CRP) level. Albumin was expressed in g/dL, lymphocyte count in cells/µL, and CRP in mg/dL for all calculations (18).

Preoperative laboratory measurements for CRP, serum albumin, and lymphocyte count were obtained as part of the routine preoperative workup, using the last available values within seven days prior to surgery. The CALLY index was calculated according to the following formula (18):


$$\text{CALLY index}=\frac{\text{Serum Albumin}\left(\text{g}/\text{dL}\right)\times \text{Lymphocyte Count}\left(\text{cells}/\text{µL}\right)}{\text{CRP}\left(\text{mg}/\text{dL}\right)}$$

The discriminative ability of the CALLY index for postoperative visual hallucinations was evaluated using receiver operating characteristic (ROC) curve analysis. An optimal cut-off value of 1.12 was identified based on Youden’s index. The detailed diagnostic performance measures are provided in **Supplementary Table 1** in **Supplementary File 1**, and the ROC curve is shown in **Supplementary Figure 1** in **Supplementary File 1**.

### Data collection procedure

Data were collected prospectively between September 2022 and May 2025 from cardiac surgery centers in the West Bank of Palestine. Eligible patients were approached during the preoperative period, and informed consent was obtained from all participants prior to enrollment. As part of the routine surgical workup, laboratory values for CRP, serum albumin, and lymphocyte count were obtained within 7 days before surgery, in line with the parameters of the CALLY index (19). Across all participating centers, VH was operationally defined as patient-reported perceptual experiences occurring in the absence of external stimuli, as captured by the QPE framework. The trained research team conducted QPE-based patient interviews once daily between postoperative days 4 and 7 at standardized morning timepoints, between 08:00 and 10:00, to ensure consistency across centers (16).

In addition to the presence of hallucinations, VH severity was quantified based on QPE-derived responses and incorporated into the analysis. Other hallucination characteristics, including duration and associated distress, were not systematically assessed or extracted for analysis in this study. Formal diagnostic confirmation based on DSM-5 or ICD-10 criteria was not performed, and standardized delirium assessments, such as CAM-ICU, were not conducted as part of the study protocol. Accordingly, VH was analyzed as a structured questionnaire-based postoperative perceptual outcome rather than as a formal psychiatric diagnosis or a delirium-defined endpoint. All assessors completed structured training led by consultant psychiatrists, focusing on standardized QPE administration, recognition of hallucination phenomena, and interview technique. Training included supervised practice sessions and standardized case scenarios to enhance consistency across raters. While formal inter-rater reliability coefficients were not calculated, these procedures were implemented to promote consistency across study sites.

Additional demographic and clinical variables were extracted from patients’ EMRs. All responses and clinical data were systematically documented and stored in a secure, password-protected database accessible only to the research team.

The follow-up window for the primary endpoint extended through postoperative day 7, with administrative censoring at day 7 for time-to-event analyses. This timeframe was selected to capture early inpatient postoperative VH during the period when acute perioperative inflammatory, metabolic, medication-related, and ICU-related factors are most clinically relevant and when standardized daily assessment was feasible across participating centers. Among the 1,686 patients screened, 150 were excluded because of missing follow-up or refusal to participate. In the final analytic cohort of 1,272 patients, data for the primary exposure, CALLY index, and the primary outcome, VH through postoperative day 7, were complete.

### Statistical analysis

Continuous variables were summarized as mean ± SD or median (Q1–Q3), and categorical variables as n (%). Group comparisons (<1.12 vs. ≥1.12 CALLY) used t-tests or Wilcoxon rank-sum tests for continuous variables and χ² or Fisher’s exact tests for categorical variables. Diagnostic performance of CALLY for postoperative visual hallucinations was assessed with ROC analysis, reporting area under the curve (AUC), 95% CIs, and optimal cut-off by Youden’s index; bootstrap resampling (1,000 iterations) was used for internal validation. Time-to-event outcomes were modeled with Cox regression, using restricted cubic splines with three knots to explore non-linearity. Multivariable Cox regression models were adjusted for clinically relevant covariates available across all participating centers, including age, sex, BMI, EuroSCORE II, surgery type, CPB time, LVEF, and postoperative ventilation duration. Additional baseline and perioperative variables, including renal impairment, NYHA class, redo surgery, on-pump/off-pump status, aortic cross-clamp time, vasopressor/inotrope use, and opioid analgesic exposure, were summarized descriptively and evaluated in univariable analyses where appropriate (8–10, 20). Prior psychiatric history beyond documented psychotic problems, substance-use variables other than smoking, and formal preoperative cognitive impairment assessments were not consistently available across all participating centers; therefore, these variables could not be incorporated into the primary or extended multivariable models and were addressed as potential sources of residual confounding. A sensitivity analysis was performed using an extended multivariable Cox model including additional clinical and perioperative covariates. Model assumptions were verified with Schoenfeld residuals, variance inflation factors (VIF), and residual diagnostics; sensitivity analyses yielded consistent results. Missing data were minimal (<3% across variables). For covariates with missing values, mean imputation was applied for continuous variables and mode imputation for categorical variables. The primary exposure (CALLY index) and outcome (visual hallucinations) were complete for all included patients; therefore, analyses for these variables were conducted on a complete-case basis. Analyses were performed in Stata 17 (StataCorp). A two-sided p value <0.05 was considered statistically significant. See **Supplementary Appendix 1** in **Supplementary File 1** for full statistical details. Detailed diagnostic performance measures are presented in **Supplementary Table 1** in **Supplementary File 1**, and the ROC curve is provided in **Supplementary Figure 1** in **Supplementary File 1**.

### Ethical considerations

Ethical approval was obtained from the Institutional Review Board of An-Najah National University, West Bank, Palestine (Med. Sep. 2022/8). We have obtained written informed consent from all the participants, confidentiality has been maintained, and all data have been anonymized. The study complied with the Declaration of Helsinki.

## Results

**Table 1** presents baseline characteristics stratified by the CALLY index (<1.12 vs. ≥1.12), showing 524 patients (41.2%) in the low group and 748 (58.8%) in the high group, with no major differences in age, sex, diabetes, hyperlipidemia, or most comorbidities. The low CALLY group, however, had slightly higher BMI and platelet counts, worse left ventricular ejection fraction, and more frequent use of pethidine and on-pump surgery.

**Table 1:** Baseline characteristics by CALLY index groups.

		CALLY index groups		
Variable	Category	Low group < 1.12 n = 524	High group ≥ 1.12 n = 748	P value
Demographic data				
Age, y	Mean ± SD	59.24 ± 11.01	58.24 ± 10.83	0.1082^a^
Sex, n (%)	Female	131 (25.0%)	188 (25.1%)	0.957^b^
BMI, kg/m^2^	Mean ± SD	24.28 ± 5.08	23.81 ± 3.27	0.0435^a^
Medical History				
Diabetes, n (%)	Yes	245 (46.76%)	347 (46.39%)	0.898^b^
Hyperlipidemia, n (%)	Yes	183 (34.92%)	250 (33.42%)	0.578^b^
Renal impairment, n (%)	Yes	10 (1.91%)	23 (3.07%)	0.198^b^
Smoker, n (%)	Yes	51 (9.7%)	80 (10.7%)	0.578^b^
NYHA class, n (%)	1	462 (88.17%)	641 (85.70%)	0.094^b^
	2	47 (8.97%)	63 (8.42%)	
	3	13 (2.48%)	38 (5.08%)	
	4	2 (0.38%)	6 (0.80%)	
Redo surgery, n (%)	Yes	33 (6.30%)	42 (5.61%)	0.611^b^
Type of Surgery, n (%)	CABG	384 (73.28%)	567 (75.80%)	0.309^b^
	Valve surgery	140 (26.72%)	181 (24.20%)	
EuroSCORE II	Median(Q1-Q3)	3.29 (3.16–3.29)	3.19 (2.98–3.29)	0.176^c^
Laboratory				
Hemoglobin, g/dL	Mean ± SD	11.49 ± 0.85	11.58 ± 0.92	0.098^a^
Absolute neutrophil count, cells/µL	Mean ± SD	21.94 ± 11.37	22.50 ± 13.81	0.441^a^
Platelet count, K/μL	Mean ± SD	136.72 ± 49.63	125.57 ± 53.62	<0.001^a^
Creatinine, mg/dL	Median(Q1-Q3)	0.97 (0.95–1.21)	0.99 (0.98–1.26)	0.367^c^
Calcium, mg/dL	Median(Q1-Q3)	8.54 (8.48–8.56)	8.56 (8.48–8.66)	0.285^c^
Medication use				
Adrenalin, n (%)	Yes	265 (50.57%)	356 (47.59%)	0.296^b^
Noradrenalin, n (%)	Yes	341 (65.08%)	471 (62.97%)	0.441^b^
Dobutamine, n (%)	Yes	20 (3.82%)	20 (2.67%)	0.250^b^
Pethidine, n (%)	Yes	192 (36.64%)	232 (31.02%)	0.036^b^
Morphine, n (%)	Yes	220 (41.98%)	320 (42.78%)	0.777^b^
Procedural parameters				
Pump	Off, n (%)	27 (5.15%)	69 (9.22%)	0.007^b^
	On, n (%)	497 (94.85%)	679 (90.78%)	
CPB time, min	Mean ± SD	115.36 ± 44.62	118.57 ± 44.45	0.205^a^
Aortic cross-clamp time, min	Mean ± SD	83.72 ± 40.15	83.57 ± 36.57	0.945^a^
LVEF, %	Mean ± SD	51.73 ± 6.80	52.91 ± 6.36	0.001^a^
Postoperative mechanical ventilation duration, hour	Median(Q1-Q3)	5.48 (4.00–6.00)	5.48 (4.00–6.00)	0.3744^c^

Values are presented as mean ± standard deviation (SD), median (Q1–Q3), or n (%) as appropriate. ^a^t test, ^b^Chi-square test (or Fisher’s exact test when appropriate), ^c^Wilcoxon rank-sum (Mann–Whitney) test. BMI, body mass index; NYHA, New York Heart Association; CABG, coronary artery bypass grafting; EuroSCORE II, European System for Cardiac Operative Risk Evaluation II; CPB, Cardiopulmonary Bypass; LVEF, left ventricular ejection fraction.

**Supplementary Figure 2** in **Supplementary File 1** presents Kaplan–Meier failure curves showing the 7-day cumulative incidence of postoperative visual hallucinations by CALLY Index group: Low CALLY (<1.12; n=524) vs. High CALLY (≥1.12; n=748). The y-axis displays cumulative incidence; the x-axis shows days after surgery. The table below reports the number at risk in each group at days 0–7. Between-group differences were assessed using the log-rank test (p<0.001).

In the univariable analysis of 1,272 patients (150 events), most baseline and perioperative factors were not associated with postoperative visual hallucinations; only EuroSCORE II showed a significant independent association with higher risk (HR = 1.05, 95% CI 1.02–1.09, P = 0.001), while BMI, renal impairment, and CPB time showed borderline trends. Full results are provided in **Supplementary Table 2** in **Supplementary File 1**.

**Table 2** shows that patients in the high CALLY group (≥1.12) had a significantly lower risk of postoperative visual hallucinations than those in the low group (<1.12) (HR = 0.39, 95% CI 0.28–0.54, P< 0.001), and this association remained robust after multivariable adjustment (HR = 0.38, 95% CI 0.27–0.53, P< 0.001). The full adjusted model is detailed in **Supplementary Table 3** in **Supplementary File 1**.

**Table 2:** Multivariable Cox regression including CALLY index groups and clinical covariates for the risk of postoperative visual hallucinations.

CALLY index groups	Postoperative visual hallucination (n=1272, events=150)	
	HR (95% CI)	P value
Crude		
Low	Ref	
High	0.39 (0.28–0.54)	<0.001
Adjusted ^a^		
Low	Ref	
High	0.38 (0.27–0.53)	<0.001

^a^Adjusted for: age, sex, BMI, EuroSCORE II, type of surgery (CABG vs. valve), CPB time, LVEF, and postoperative ventilation hours.

**Table 3** presents Restricted cubic spline modeling (3 knots at the 10th, 50th, and 90th percentiles) showed a significant non-linear association between the CALLY Index and hallucination risk (rcs1 HR = 0.58, p=0.003; rcs2 HR = 2.08, p=0.023). Higher EuroSCORE II remained a significant risk factor (HR = 1.05, p=0.002), Higher BMI showed borderline protection (HR = 0.95, p=0.048), while other covariates were not significant.

**Table 3:** Multivariable Cox regression including restricted cubic spline (3 knots) for CALLY Index and risk of postoperative visual hallucinations (N = 1,272; 150 events).

Variable	HR (95% CI)	p-value
CALLY Index (RCS, 3 knots)		
– rcs1	0.58 (0.40–0.83)	0.003
– rcs2	2.08 (1.11–3.91)	0.023
Age (per year)	1.00 (0.99–1.02)	0.600
Female sex	1.18 (0.81–1.73)	0.385
BMI (per kg/m²)	0.95 (0.91–1.00)	0.048
EuroSCORE II (per unit)	1.05 (1.02–1.08)	0.002
Valve surgery (vs CABG)	0.93 (0.61–1.41)	0.736
CPB time (per min)	1.00 (0.99–1.00)	0.216
LVEF (per %)	1.02 (0.99–1.04)	0.226
Postoperative ventilation (per hour)	0.97 (0.93–1.02)	0.237

HR, Hazard Ratio; CI, Confidence Interval; RCS, Restricted Cubic Spline; BMI, Body Mass Index; EuroSCORE II, European System for Cardiac Operative Risk Evaluation II; CABG, Coronary Artery Bypass Grafting; CPB, Cardiopulmonary Bypass; LVEF, Left Ventricular Ejection Fraction.

**Figure 2:**
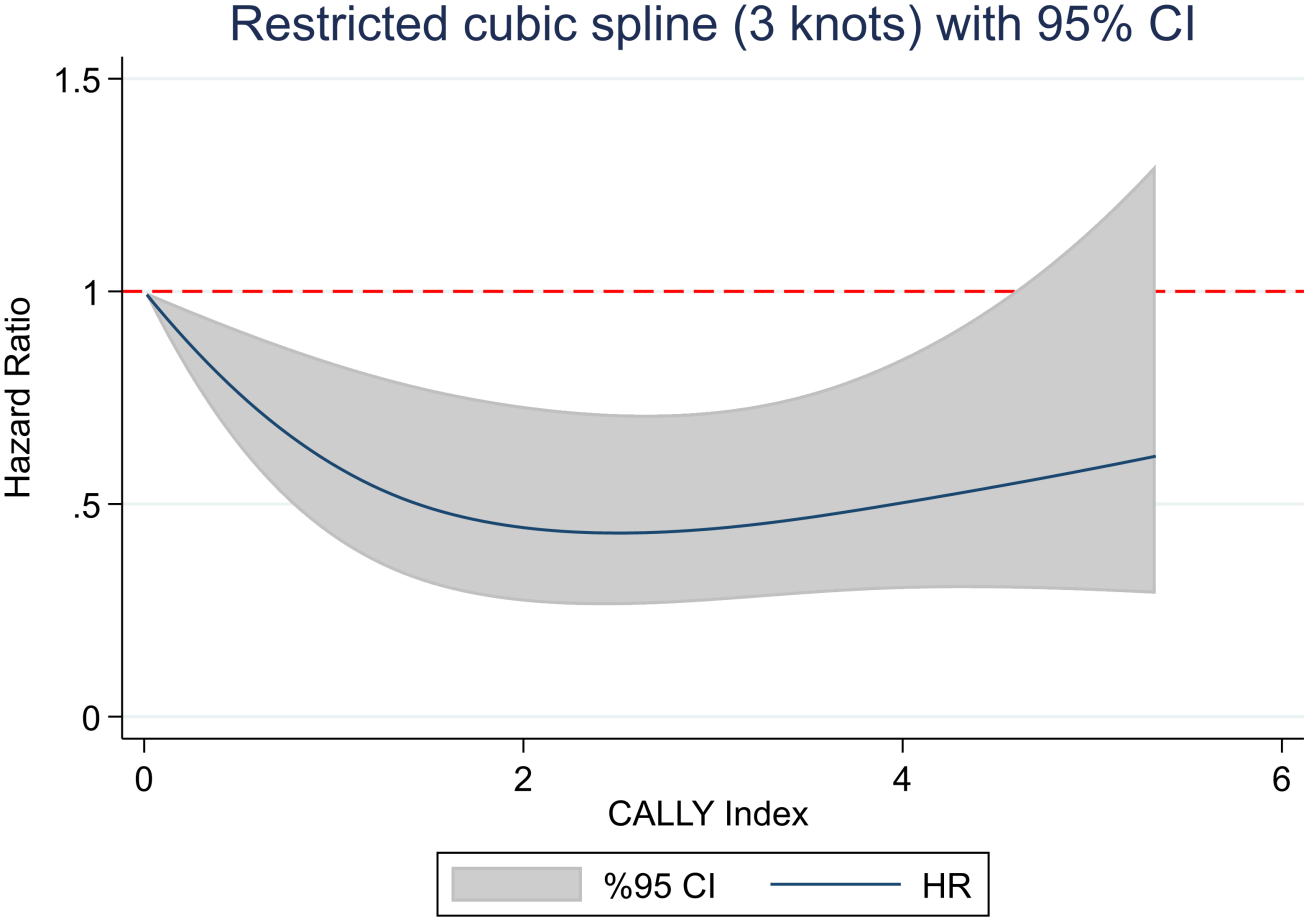
Restricted cubic spline plot of the association between CALLY Index and risk of postoperative visual hallucinations.

Restricted cubic spline analysis demonstrated a non-linear association between the CALLY index and postoperative visual hallucination risk, as shown in **Figure 2**. The solid line depicts the estimated hazard ratio across the observed range of CALLY Index values, while the shaded area represents the 95% confidence interval. The dashed line at HR = 1 denotes the reference level. The estimated hazard decreased from low to moderate CALLY values, reaching its lowest point in the mid-range of the distribution, and then appeared to rise again at higher CALLY values. Estimates at the upper end of the CALLY distribution should be interpreted cautiously because the confidence intervals widened, suggesting lower precision in this range.

**Figure 3:**
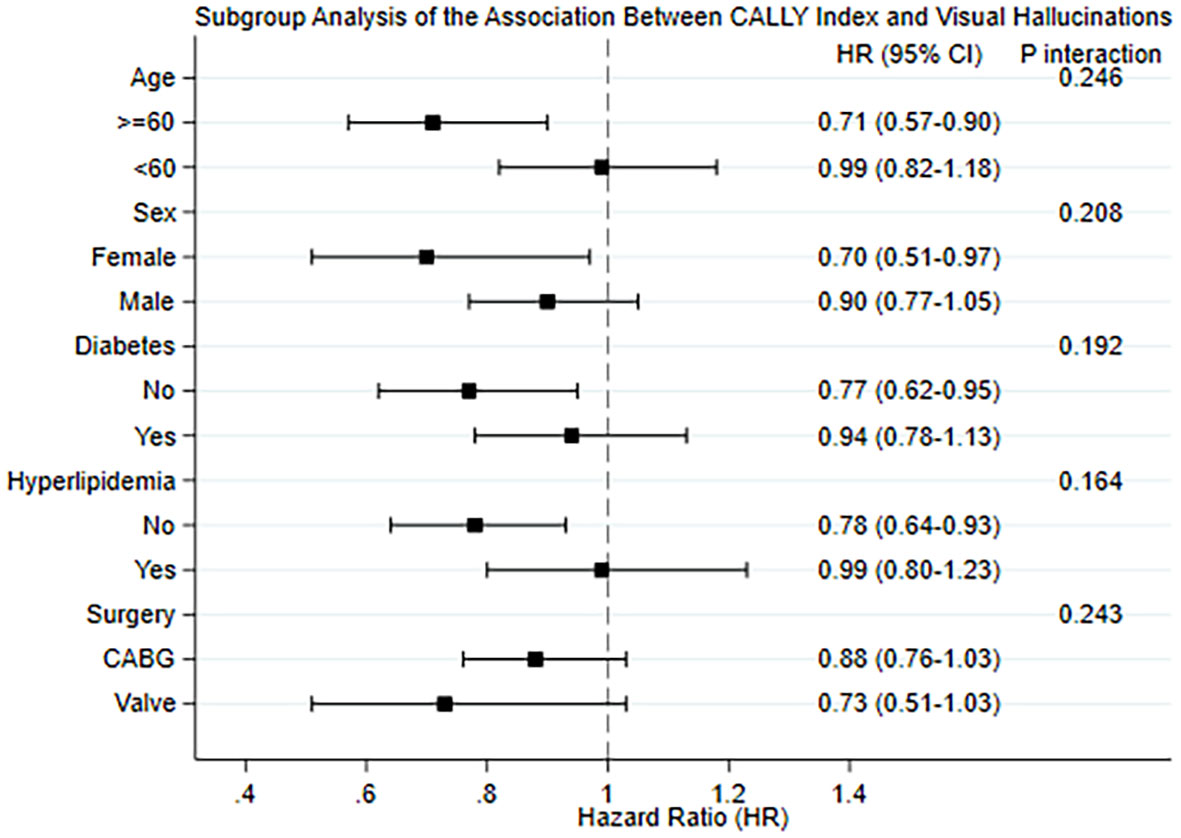
Subgroup analysis of the association between CALLY index and visual hallucinations.

**Figure 3** presents the stratified subgroup analysis of the association between the CALLY index and postoperative visual hallucinations. Higher CALLY values were generally associated with a lower hazard of visual hallucinations across the predefined clinical subgroups, although the magnitude and statistical significance of the estimates varied. The association appeared significant among patients aged ≥60 years (HR 0.71, 95% CI 0.57–0.90), females (HR 0.70, 95% CI 0.51–0.97), non-diabetic patients (HR 0.77, 95% CI 0.62–0.95), and patients without hyperlipidemia (HR 0.78, 95% CI 0.64–0.93). In contrast, estimates were attenuated and not statistically significant among patients aged<60 years, males, diabetic patients, patients with hyperlipidemia, and across surgery-type strata. However, formal interaction testing did not demonstrate statistically significant effect modification by age, sex, diabetes, hyperlipidemia, or surgery type (all p for interaction >0.05). Therefore, these subgroup findings should be interpreted as exploratory and hypothesis-generating rather than evidence of differential treatment or prognostic effects across subgroups.

To further characterize hallucinations, we analyzed QPE-derived visual hallucination severity. As shown in **Supplementary Table 4**, there was no significant difference in severity between CALLY index groups (median 3 [IQR 1–6] vs. 3 [IQR 1–7], p=0.41). In multivariable linear regression analysis adjusted for age, sex, BMI, and EuroSCORE II, CALLY group was not associated with hallucination severity (β=0.33, 95% CI −0.89 to 1.56, p=0.592).

To further address potential residual confounding, we performed an extended multivariable Cox regression model including additional clinical and perioperative variables. As shown in **Supplementary Table 5**, the association between the CALLY index and postoperative visual hallucinations remained robust, with patients in the high CALLY group demonstrating a significantly lower risk compared with the low CALLY group (HR 0.37, 95% CI 0.26–0.52, p<0.001).

## Discussion

Our study shows that the CALLY Index, a composite of CRP, albumin, and lymphocyte count, is a strong early predictor of postoperative visual hallucinations after cardiac surgery. In 1,272 patients, higher CALLY was linked to lower risk in both crude and adjusted models, with early divergence on Kaplan–Meier curves and a significant non-linear association on spline analysis. The effect was most evident in older patients, women, non-diabetics, and those without hyperlipidemia. To our knowledge, this is the first study to define a cardiac surgery–specific cut-off (1.12), underscoring CALLY’s value as a practical, routinely available risk stratification tool beyond oncology.

From a clinical perspective, the magnitude of the adjusted association suggests that patients with lower preoperative CALLY values represent a higher-risk subgroup who may benefit from closer early postoperative monitoring for VH, a clinically relevant outcome associated with postoperative distress and adverse recovery trajectories (5, 7). However, the diagnostic performance of CALLY should be interpreted conservatively. In the present study, the ROC analysis showed moderate discrimination, and the selected cut-off demonstrated a high negative predictive value but a low positive predictive value, as shown in **Supplementary Table 1**. Therefore, CALLY may be most useful as a simple adjunctive risk-stratification marker rather than as a standalone diagnostic or decision-making tool. Its clinical value lies in complementing, not replacing, comprehensive perioperative assessment and postoperative neuropsychiatric monitoring.

Interestingly, although the CALLY index was strongly associated with the occurrence of postoperative visual hallucinations, it was not associated with their severity. This finding suggests that the biological pathways captured by the CALLY index, namely systemic inflammation, nutritional status, and immune competence, may primarily influence susceptibility to developing hallucinations rather than the intensity of the perceptual disturbance once it occurs. Clinically, this distinction supports the role of CALLY as a risk stratification tool for identifying vulnerable patients, rather than as a marker of symptom burden or progression. Importantly, the stability of the association in the extended multivariable model supports the robustness of the findings and suggests that the observed relationship is unlikely to be fully explained by residual confounding.

Mechanistically, the association between lower CALLY and VH risk may reflect the combined effects of systemic inflammation, reduced nutritional reserve, and impaired immune competence on postoperative brain vulnerability. A lower CALLY index reflects one or more of the following: elevated CRP, reduced serum albumin, or decreased lymphocyte count, which correspond to high inflammatory status, poor nutritional state, and impaired immune function, respectively. Each of these components has been independently associated with adverse postoperative outcomes (9, 13), and their combination may contribute to increased vulnerability to neuropsychiatric complications such as hallucinations.

This study is the first to identify a CALLY index cut-point of 1.12 for cardiac surgery patients, showing its prognostic value for postoperative hallucinations. This is lower than oncology cohorts, where cut-points are higher, 3.0 in ovarian cancer (21), 2.285 in breast cancer (20), and generally 2.0–2.3 in other tumors (9, 20, 22, 23), reflecting differing inflammatory and nutritional profiles across populations.

In oncology patients, the CALLY index reflects systemic inflammation, nutrition, and immune status amid cancer-related cachexia and chronic inflammation (24), leading to higher CRP, lower albumin/lymphocytes, and higher risk thresholds (25). Cardiac surgery patients face mainly acute inflammation (26) and, despite common comorbidities, usually lack cachexia or prolonged systemic inflammation (27).

The surgical setting itself further distinguishes this group. Cardiac surgery, particularly procedures involving cardiopulmonary bypass, induces a strong systemic inflammatory response through mechanisms such as reperfusion injury and contact activation of blood components (28). This acute inflammatory surge can precipitate a range of complications, including neuropsychiatric outcomes like delirium and hallucinations (28, 29).

Importantly, delirium and related hallucinations occur in up to one-third of cardiac surgery patients, far exceeding the incidence seen in most non-cardiac elective operations (27, 30, 31). These events are not only distressing but are also associated with prolonged hospital stay, long-term cognitive decline, and increased mortality (32). In our study, 414 patients were excluded to ensure the validity of the CALLY index in the cardiac surgical context, specifically those with hematologic or immune disorders, corticosteroid therapy, active malignancy or infection, and severe malnutrition (33). These conditions profoundly alter serum albumin, CRP, and lymphocyte counts. The components of the index, through mechanisms such as chronic immune dysregulation (31), corticosteroid-induced lymphopenia and hypoalbuminemia (34, 35), or cancer- and infection-related systemic inflammation and cachexia, which distort biomarker distribution and necessitate higher prognostic cut-offs in oncology cohorts (23, 32, 36). Severe malnutrition was also excluded, as hypoalbuminemia here reflects chronic nutritional depletion rather than perioperative stress, and is independently linked to higher rates of delirium, infection, and mortality after cardiac surgery (8, 24). Notably, serum albumin serves not only as a nutritional marker but also as an antioxidant, transporter, and regulator of oncotic pressure and vascular integrity; low levels, whether due to malnutrition, liver disease, or inflammation, are consistently associated with reduced reserve and poor postoperative outcomes (27).

C-reactive protein (CRP) is a key biomarker in cardiac surgery, with elevated levels reflect systemic inflammation that may worsen during cardiopulmonary bypass, disrupt the blood–brain barrier, activate microglia, and impair neuronal function, thereby increasing the risk of postoperative delirium (POD) and postoperative cognitive dysfunction (POCD) (34, 37). High perioperative CRP is linked to delirium in older patients, longer ICU stay, and cognitive decline, with peaks on days 2–3 after CABG associated with complications (30, 31). Collectively, CRP predicts adverse neurocognitive outcomes (38).

Research indicates that low preoperative albumin levels can predict POD and cognitive issues. Severe hypoalbuminemia (≤30 g/L) is linked to higher POD risk in elderly surgical patients, and low albumin levels predict delirium in elective cardiac surgeries (39–41). Reduced preoperative albumin also serves as a strong independent predictor of delirium in cardiac ICU patients (39). Preoperative lymphopenia is a robust predictor of adverse neurocognitive outcomes after cardiac surgery, reflecting impaired adaptive immunity and heightened systemic stress responses (42). Lymphocytes regulate peripheral inflammation and support blood–brain barrier integrity; their depletion permits unchecked innate immune activation, microglial priming, and neurotransmitter imbalance, mechanisms implicated in delirium and hallucinations (42, 43). Within the CALLY index, reduced lymphocyte counts directly lower the score, capturing this immunological vulnerability and linking it to increased postoperative neuropsychiatric risk.

Composite hematologic indices that integrate multiple immune components have demonstrated added predictive value across cardiovascular settings. Ratios such as the neutrophil-to-lymphocyte ratio (NLR), platelet-to-lymphocyte ratio (PLR), and monocyte-to-lymphocyte ratio (MLR) capture the interplay between pro-inflammatory and adaptive immune responses. For example, in patients undergoing trans-catheter aortic valve replacement (TAVR), elevated NLR has been shown to predict adverse outcomes such as the need for permanent pacemaker implantation, underscoring the broader prognostic role of these immune-inflammatory markers in cardiac interventions (44).

Elevated values consistently reflect systemic inflammation coupled with impaired immune surveillance, and these indices have been linked to a higher incidence of postoperative delirium across diverse surgical populations, including orthopedic, esophageal, and head and neck procedures (45–47). More sophisticated markers, such as the systemic immune-inflammation index (SII) and systemic inflammation response index (SIRI), have also demonstrated predictive ability for hyperactive delirium in intensive care and perioperative settings (48, 49). Importantly, similar immune-inflammatory dynamics have been observed in trans-catheter aortic valve replacement (TAVR), where elderly patients exhibited pronounced increases in neutrophil-to-lymphocyte ratio (NLR) and neutrophil counts, which were associated with higher early complications and mortality, reinforcing the prognostic role of systemic inflammation in cardiovascular interventions (50).

The restricted cubic spline analysis suggests that the association between the CALLY index and postoperative visual hallucinations is not strictly linear. Although the categorical analysis supports low CALLY as a marker of increased risk, the continuous spline model indicates that the lowest estimated risk occurs at moderate CALLY values, with a possible rise in risk at higher values. This finding suggests that CALLY should not be interpreted as a biomarker for which progressively higher values are uniformly protective.

Several mechanisms may explain this pattern. First, very high CALLY values may reflect disproportionately elevated albumin levels, which can be influenced by hemoconcentration or perioperative fluid shifts rather than true nutritional advantage. Such states may indicate physiological stress and altered vascular dynamics that could contribute to neuropsychiatric vulnerability. Second, low preoperative CRP does not preclude a strong postoperative inflammatory response. Cardiac surgery, particularly with cardiopulmonary bypass, can induce acute systemic inflammation, endothelial dysfunction, and blood–brain barrier disruption, all of which have been implicated in postoperative delirium and related perceptual disturbances (51, 52). Third, lymphocyte counts may not fully represent immune competence; perioperative stress can lead to immune redistribution and dysregulation, suggesting that both immune suppression and dysregulated immune activation may be associated with adverse neuropsychiatric outcomes (51).

Finally, the apparent increase in risk at higher CALLY values may partly reflect statistical instability, as fewer patients contribute data at the extremes of the distribution and confidence intervals are wider. Therefore, this upper-range pattern should be interpreted cautiously and considered hypothesis-generating rather than definitive.

Subgroup analyses suggested stronger protection in patients ≥60 years, women, and those without diabetes or hyperlipidemia, while effects were weaker in younger, male, or metabolically comorbid patients. These differences may reflect true effect modification or limited precision, but the overall direction was consistent. Findings highlight the biological plausibility of low CALLY as a marker of catabolic stress and inflammation, though subgroup results remain exploratory and need validation.

Although the association between lower CALLY index and postoperative visual hallucinations remained robust after adjustment for major demographic, surgical, and clinical covariates, residual confounding cannot be excluded. Several factors known to influence postoperative neuropsychiatric outcomes, including detailed sedative and anticholinergic medication exposure, anesthesia depth, sleep disruption, physical restraints, ICU environmental stressors, and formal preoperative cognitive or psychiatric status, were not consistently available across centers. Available variables such as EuroSCORE II, cardiopulmonary bypass time, postoperative ventilation duration, vasopressor/inotrope use, and opioid analgesic exposure partially reflect perioperative illness severity and treatment burden but do not fully capture these unmeasured domains.

## Limitations

This study is limited by its 7-day follow-up, single time-point CALLY measurement, and selective exclusions, which may restrict causal inference and generalizability. Because VH was assessed only during the early postoperative period through day 7, delayed or persistent hallucinations occurring after this window may have been missed. Therefore, the findings should be interpreted as applying to early postoperative VH rather than to the full burden of hallucinations during later recovery. Residual confounding remains an important limitation of this observational study. Detailed data on sedative and anticholinergic exposure, anesthesia depth, ICU sleep disruption, physical restraints, prior psychiatric history beyond documented psychotic problems, substance-use variables other than smoking, and formal preoperative cognitive impairment assessment**s** were not systematically collected across all centers. In addition, although the multicenter design improves clinical relevance within the study region, center-level heterogeneity in perioperative care pathways, ICU environments, laboratory workflows, medication practices, and neuropsychiatric assessment implementation may have influenced outcome ascertainment and residual confounding. Because center-level random effects or frailty terms were not incorporated, this potential source of heterogeneity could not be formally quantified. Therefore, the observed association between CALLY index and postoperative visual hallucinations should be interpreted as prognostic rather than causal. Although predefined exclusion criteria were applied to improve internal validity, these may have introduced selection bias by yielding a more clinically homogeneous cohort.

The findings of this study should be interpreted in the context of a geographically and clinically selected population. The cohort consisted of patients undergoing cardiac surgery across centers in the West Bank, Palestine, and excluded individuals with malignancy, active infection, and severe malnutrition. As a result, the study population represents a relatively homogeneous surgical cohort rather than a general population. These selection criteria may limit the external validity of the findings, particularly in settings with different patient profiles, healthcare systems, or baseline inflammatory and nutritional characteristics. Although similar associations may be biologically plausible in other perioperative or critical illness settings because the CALLY index reflects inflammation, nutritional reserve, and immune competence, the present findings should not be assumed to apply directly to non-cardiac surgery populations or non-surgical populations. Procedure-specific factors in cardiac surgery, including cardiopulmonary bypass exposure, acute perioperative inflammation, ICU-related stressors, and postoperative medication patterns, may influence both CALLY dynamics and VH risk. Therefore, the observed association, the 1.12 cut-off, and the clinical utility of CALLY require external validation in non-cardiac surgical cohorts, other hospitalized populations, and different healthcare settings before broader application. In addition, while missing data were minimal and addressed using simple imputation methods, residual bias cannot be fully excluded. Therefore, the findings should be interpreted with consideration of potential selection and information bias inherent to observational study designs.

Data-driven thresholds and exploratory subgroups also require external validation. Hallucinations were assessed using a structured questionnaire (QPE) without formal diagnostic confirmation based on DSM-5 or ICD-10 criteria. In addition, standardized delirium assessments (e.g., CAM-ICU) were not performed; therefore, we could not definitively distinguish isolated hallucinations from those occurring in the context of delirium. This limitation may have introduced outcome misclassification and limits clinical interpretability. Although structured assessor training, standardized interview procedures, supervised practice sessions, standardized case scenarios, and uniform postoperative assessment timing were used to promote consistency across centers, formal inter-rater reliability coefficients were not calculated. Therefore, residual between-assessor or between-center variability in VH assessment cannot be excluded and may have contributed to information bias. Furthermore, detailed phenomenological characteristics such as duration and distress were not systematically assessed within the scope of the study.

Although several validated multivariable models exist for predicting postoperative delirium after cardiac surgery, direct comparison with these models was not feasible in the present study. This is primarily due to differences in outcome definition, as the current analysis focused on visual hallucinations rather than delirium, as well as the absence of key variables required to reconstruct established prediction scores, including detailed neurocognitive assessments and perioperative medication profiles. Accordingly, the CALLY index should not be interpreted as a replacement for comprehensive multivariable prediction models but rather as a simple, biomarker-based tool that may complement existing approaches. Given its derivation from routine laboratory parameters, CALLY offers practical advantages in terms of accessibility and ease of implementation. Future studies should evaluate the incremental predictive value of CALLY when integrated into established delirium prediction models using formal comparative metrics such as AUC, NRI, and IDI.

Future work should extend follow-up, incorporate repeated CALLY measures, and test perioperative strategies (e.g., nutritional support, anti-inflammatory bundles, immune-preserving protocols) in multicenter trials.

## Conclusion

In this multicenter cohort of cardiac surgery patients, the preoperative CALLY Index was associated with early postoperative visual hallucinations. These findings support its potential role as a practical risk stratification tool in similar clinical settings; however, external validation in diverse populations is required before broader application.

## Data Availability

The raw data supporting the conclusions of this article will be made available by the authors, without undue reservation.
