# Supplementary material for: Prognostic value of the C-reactive protein–albumin–lymphocyte index for postoperative visual hallucinations following cardiac surgery
Source: Front Psychiatry. 2026 Jul 24;17:1729831. doi: 10.3389/fpsyt.2026.1729831 (PMC13447496; doi:10.3389/fpsyt.2026.1729831)
Supplement: Supplementary file 1 [file Supplementaryfile1.docx]

**Supplementary File 1**

**Supplementary Graphical Abstract**

**Supplementary Appendix 1. Statistical analysis**

**Supplementary Table 1. Diagnostic performance of the CALLY index at the optimal cut-off (1.12) for postoperative visual hallucinations**

**Supplementary Figure 1.** **Receiver operating characteristic (ROC) curve of the CALLY index for predicting postoperative visual hallucinations.**

**Supplementary Figure 2. Kaplan–Meier curves by CALLY Index group (follow-up to day 7)**

**Supplementary Table 2. Time-to-event analysis for postoperative Visual hallucinations (n=1272, events=150)**

**Supplementary Table 3. Multivariable Cox regression including CALLY index groups and clinical covariates for the risk of postoperative visual hallucinations.**

**Supplementary Table 4. Severity of postoperative visual hallucinations according to CALLY index groups**

**Supplementary Table 5. Extended multivariable Cox regression model including additional clinical and perioperative covariates**

**Supplementary Graphical Abstract**

**Supplementary Appendix 1. Statistical analysis**

Continuous variables were summarized as mean ± standard deviation (SD) when approximately normally distributed, and as median (Q1–Q3) when skewed or containing outliers. Categorical variables were presented as n (%). Between the two CALLY Index groups (<1.12 vs. ≥1.12), we compared continuous variables using independent-samples t-tests after visually checking normality and, where appropriate, equality of variances; when these assumptions were not met, we used the Wilcoxon rank-sum (Mann–Whitney) test. Categorical variables were compared using χ² tests; if any expected cell count was <5, we used Fisher’s exact test. All tests were two-sided with α=0.05.

To assess the ability of the CALLY Index to discriminate between patients who developed postoperative visual hallucinations and those who did not, we performed receiver operating characteristic (ROC) analysis and reported the area under the curve (AUC) along with 95% confidence intervals (CIs). Because lower CALLY values were hypothesized to indicate higher risk, we analyzed the inverse of CALLY to ensure the ROC curve had the correct directionality. The optimal cut-off was determined by maximizing Youden’s index (sensitivity + specificity − 1). At that threshold, we reported sensitivity, specificity, positive and negative predictive values (PPV/NPV), and likelihood ratios (LR+ and LR−). Internal validation employed bootstrap resampling (1,000 iterations) to obtain an optimism-corrected AUC and to assess the stability of the selected cut-off.

We modeled time from surgery to the first occurrence of visual hallucinations using Cox proportional hazards regression with administrative censoring at day 7. The CALLY Index was entered as a continuous predictor using restricted cubic splines (RCS) with three knots placed at the 10th, 50th, and 90th percentiles of its empirical distribution. We set the median CALLY as the reference value for interpretation and plotting. We reported hazard ratios (HRs) with 95% CIs. Departure from linearity was tested with a likelihood-ratio test comparing the spline model to a linear-term model. To aid interpretation, we plotted the adjusted HR across the observed CALLY range, with a shaded 95% CI band and a reference line at HR = 1 (Figure 2).To evaluate the association between the CALLY Index and hallucination risk, we adjusted for confounding based on clinical relevance and prior literature (8–10,19), Multivariable Cox regression models were adjusted for clinically relevant covariates available across all participating centers, including age, sex, BMI, EuroSCORE II, surgery type, cardiopulmonary bypass time, left ventricular ejection fraction, and postoperative ventilation duration. Additional baseline and perioperative variables, including renal impairment, NYHA class, redo surgery, on-pump/off-pump status, aortic cross-clamp time, vasopressor/inotrope use, and opioid analgesic exposure, were summarized descriptively and evaluated in univariable analyses where appropriate.

The proportional hazards assumption was assessed with scaled Schoenfeld residuals both globally and for each covariate; no meaningful violations were detected. Multicollinearity was examined using variance inflation factors (VIF) and found to be low (all <5). Influence and fit were explored using Martingale and deviance residuals; no observations had an undue influence on the results. We conducted sensitivity analyses by (i) fitting a model with CALLY as a linear term, (ii) removing individual covariates one at a time, and (iii) restricting to complete follow-up through day 7; conclusions were unchanged. Missing data were minimal (<3% across variables). For covariates with missing values, mean imputation was applied for continuous variables and mode imputation for categorical variables. The primary exposure (CALLY index) and outcome (visual hallucinations) were complete for all included patients; therefore, analyses for these variables were conducted on a complete-case basis. All analyses were performed in Stata 17 (StataCorp LLC, College Station, TX, USA). Two-sided p-values <0.05 were considered statistically significant.

**Supplementary Table 1. Diagnostic performance of the CALLY index at the optimal cut-off (1.12) for postoperative visual hallucinations.**

| **Metric** | **Value (95% CI)** |
| --- | --- |
| **AUC** | **0.68 (0.63–0.73)** |
| **Optimal cut-off (CALLY)** | **1.12 (Youden’s index)** |
| **Sensitivity (%)** | **70** |
| **Specificity (%)** | **65** |
| **Positive predictive value (%)** | **25** |
| **Negative predictive value (%)** | **94** |
| **LR+** | **2.0** |
| **LR–** | **0.46** |

**AUC: Area Under Curve**

**Supplementary Figure 1.** **Receiver operating characteristic (ROC) curve of the CALLY index for predicting postoperative visual hallucinations.**

**Supplementary Figure 1 presents an AUC of 0.68 (95% CI: 0.63–0.73).**

**Supplementary Figure 2. Kaplan–Meier curves by CALLY Index group (follow-up to day 7)**

**Supplementary Table 2. Time-to-event analysis for postoperative Visual hallucinations (n=1272, events=150)**

|  | | **Visual Hallucinations**  **(n=1272, events=150)** |  |
| --- | --- | --- | --- |
|  | | **HR (%95 CI)** | **P value** |
| **Variable** | **Category** |  |  |
| **Demographic data** |  |  |  |
| **Age, y** |  | 1.003 (0.988–1.018) | 0.723 |
| **Gender n, %** | **Female** | 1.168 (0.818–1.668) | 0.394 |
| **BMI, kg/m^2^** |  | 0.960 (0.916–1.005) | 0.083 |
| **Medical History** | |  |  |
| **Diabetes, n (%)** | **Yes** | 1.004 (0.729–1.384) | 0.980 |
| **Hyperlipidemia, n (%)** | **Yes** | 1.095 (0.785–1.529) | 0.594 |
| **Renal impairment, n (%)** | **Yes** | 1.951 (0.914–4.167) | 0.084 |
| **Smoker** | **Yes** | 0.755 (0.419–1.362) | 0.351 |
| **NYHA class** | **2 vs 1** | 1.438 (0.878–2.355) | 0.149  0.233  0.937 |
|  | **3 vs 1** | 0.498 (0.158–1.564) |  |
|  | **4 vs 1** | 1.083 (0.151–7.748) |  |
| **Redo surgery, n (%)** | **Yes** | 1.323 (0.716–2.444) | 0.372 |
| **Type of Surgery, n (%)** | **Valve surgery vs CABG** | 1.000 (0.692–1.445) | 1.000 |
| **EuroSCORE II,** |  | **1.053 (1.022–1.086)** | **0.001** |
| **Laboratory** | |  |  |
| **Haemoglobin, g/dL** |  | 0.991 (0.825–1.191) | 0.927 |
| **Absolute neutrophils, cells/µL** |  | 1.003 (0.991–1.015) | 0.644 |
| **Platelet, K/μL** |  | 1.002 (0.999–1.005) | 0.285 |
| **Creatinine, mg/dL** |  | 1.065 (0.875–1.297) | 0.528 |
| **Calcium, mg/dL** |  | 1.233 (0.704–2.157) | 0.464 |
| **Medication use** | |  |  |
| **Adrenalin, n (%)** | **Yes** | 0.916 (0.664–1.262) | 0.590 |
| **Noradrenalin, n (%)** | **Yes** | 1.044 (0.747–1.460) | 0.799 |
| **Dobutamin, n (%)** | **Yes** | 0.403 (0.100–1.627) | 0.202 |
| **Pethidine, n (%)** | **Yes** | 1.158 (0.831–1.614) | 0.387 |
| **Morphine, n (%)** | **Yes** | 0.981 (0.709–1.356) | 0.906 |
| **Procedural parameters** | |  |  |
| **Pump** | **On vs Off** | 0.914 (0.507–1.649) | 0.766 |
| **Cardiopulmonary bypass time, min** |  | 0.997 (0.993–1.000) | 0.081 |
| **Aortic cross clamp time, min** |  | 1.000 (0.996–1.004) | 0.912 |
| **Operating time, min** |  | 0.999 (0.996–1.001) | 0.327 |
| **Left ventricle ejection fraction, %** |  | 1.012 (0.986–1.038) | 0.365 |
| **Mechanical ventilation time postoperative, hour** |  | 0.967 (0.921–1.016) | 0.181 |

**HR = Hazard Ratio; CI = Confidence Interval; BMI = Body Mass Index; NYHA = New York Heart Association; CABG = Coronary Artery Bypass Grafting; EuroSCORE II = European System for Cardiac Operative Risk Evaluation II**

**Supplementary Table 3. Multivariable Cox regression including CALLY index groups and clinical covariates for the risk of postoperative visual hallucinations.**

| **Variable** | **HR (95% CI)** | **P value** |
| --- | --- | --- |
| **CALLY index** |  |  |
| **Low (<1.12)** | Ref |  |
| **High (≥1.12)** | 0.39 (0.28–0.54) | <0.001 |
|  |  |  |
| **Age, years** | 1.003 (0.988–1.018) | 0.722 |
| **Female sex** | 1.17 (0.80–1.72) | 0.411 |
| **BMI, kg/m²** | 0.95 (0.91–1.00) | 0.042 |
| **EuroSCORE II** | 1.05 (1.02–1.08) | 0.001 |
| **Type of surgery** |  |  |
| **Valve vs CABG** | 0.91 (0.60–1.39) | 0.674 |
| **Cardiopulmonary bypass time, min** | 0.998 (0.994–1.002) | 0.278 |
| **LVEF, %** | 1.02 (0.99–1.04) | 0.177 |
| **Post-operative ventilation, h** | 0.98 (0.93–1.02) | 0.301 |

**HR = Hazard Ratio; CI = Confidence Interval; BMI = Body Mass Index; CABG = Coronary Artery Bypass Grafting; EuroSCORE II = European System for Cardiac Operative Risk Evaluation II; LVEF: Left Ventricular Ejection Fraction**

**Supplementary Table 4. Severity of postoperative visual hallucinations according to CALLY index groups**

| **Variable** | **High CALLY (≥1.12) (n=55)** | **Low CALLY (<1.12) (n=95)** | **P value** |
| --- | --- | --- | --- |
| **VH severity, mean ± SD** | 3.78 ± 3.80 | 4.11 ± 3.59 | 0.41* |
| **VH severity, median (IQR)** | 3 (1–6) | 3 (1–7) |  |
| **Multivariable analysis (β coefficient for Low vs High)** | Reference | 0.33 (95% CI −0.89 to 1.56) | 0.592 |

*P-value from Wilcoxon rank-sum test
Multivariable model adjusted for age, sex, BMI, and EuroSCORE II

**Supplementary Table 5. Extended multivariable Cox regression model including additional clinical and perioperative covariates**

| **Variable** | **HR (95% CI)** | **P value** |
| --- | --- | --- |
| **CALLY index (High vs Low)** | **0.37 (0.26–0.52)** | **<0.001** |
| Age (per year) | 1.00 (0.99–1.02) | 0.588 |
| Female sex | 1.16 (0.79–1.70) | 0.442 |
| BMI (per kg/m²) | 0.95 (0.91–1.00) | 0.043 |
| EuroSCORE II | 1.05 (1.01–1.08) | 0.006 |
| Valve vs CABG | 0.92 (0.60–1.41) | 0.697 |
| Cardiopulmonary bypass time | 1.00 (0.99–1.00) | 0.093 |
| LVEF (%) | 1.01 (0.99–1.04) | 0.306 |
| Postoperative ventilation (h) | 0.98 (0.93–1.03) | 0.369 |
| Renal impairment | 2.27 (0.99–5.20) | 0.052 |
| NYHA class 2 vs 1 | 1.32 (0.78–2.23) | 0.299 |
| NYHA class 3 vs 1 | 0.45 (0.14–1.49) | 0.191 |
| NYHA class 4 vs 1 | 1.08 (0.15–8.02) | 0.938 |
| Redo surgery | 1.29 (0.67–2.47) | 0.445 |
| On-pump vs off-pump | 0.73 (0.39–1.38) | 0.339 |
| Cross-clamp time | 1.00 (0.998–1.01) | 0.161 |
| Adrenalin | 0.79 (0.56–1.11) | 0.171 |
| Noradrenalin | 1.04 (0.74–1.48) | 0.804 |
| Dobutamin | 0.33 (0.08–1.36) | 0.125 |
| Pethidine | 1.11 (0.76–1.62) | 0.582 |
| Morphine | 0.94 (0.65–1.35) | 0.742 |

**References**

8.	Luo L, Li M, Xi Y, Hu J, Hu W. C-reactive protein-albumin-lymphocyte index as a feasible nutrition-immunity-inflammation marker of the outcome of all-cause and cardiovascular mortality in elderly. Clin Nutr ESPEN. 2024 Oct 1;63:346–53.

9.	Wu B, Liu J, Shao C, Yu D, Liao J. Integrating inflammation, nutrition, and immunity: the CALLY index as a prognostic tool in digestive system cancers - a systematic review and meta-analysis. BMC Cancer. 2025 Apr 11;25(1):672.

10.	Lewis SR, Pritchard MW, Fawcett LJ, Punjasawadwong Y. Bispectral index for improving intraoperative awareness and early postoperative recovery in adults. Cochrane Database Syst Rev. 2019 Sept 26;2019(9):CD003843.

19.	Zhuang J, Wang S, Wang Y, Wu Y, Hu R. Prognostic Value of CRP-Albumin-Lymphocyte (CALLY) Index in Patients Undergoing Surgery for Breast Cancer. Int J Gen Med. 2024;17:997–1005.
